# Action Potential Duration Heterogeneity of Cardiac Tissue Can Be Evaluated from Cell Properties Using Gaussian Green's Function Approach

**DOI:** 10.1371/journal.pone.0079607

**Published:** 2013-11-18

**Authors:** Arne Defauw, Ivan V. Kazbanov, Hans Dierckx, Peter Dawyndt, Alexander V. Panfilov

**Affiliations:** 1 Department of Physics and Astronomy, Ghent University, Ghent, Belgium; 2 Department of Applied Mathematics and Computer Science, Ghent University, Ghent, Belgium; University of Minnesota, United States of America

## Abstract

Action potential duration (APD) heterogeneity of cardiac tissue is one of the most important factors underlying initiation of deadly cardiac arrhythmias. In many cases such heterogeneity can be measured at tissue level only, while it originates from differences between the individual cardiac cells. The extent of heterogeneity at tissue and single cell level can differ substantially and in many cases it is important to know the relation between them. Here we study effects from cell coupling on APD heterogeneity in cardiac tissue in numerical simulations using the ionic TP06 model for human cardiac tissue. We show that the effect of cell coupling on APD heterogeneity can be described mathematically using a Gaussian Green's function approach. This relates the problem of electrotonic interactions to a wide range of classical problems in physics, chemistry and biology, for which robust methods exist. We show that, both for determining effects of tissue heterogeneity from cell heterogeneity (forward problem) as well as for determining cell properties from tissue level measurements (inverse problem), this approach is promising. We illustrate the solution of the forward and inverse problem on several examples of 1D and 2D systems.

## Introduction

Cardiac contraction is initiated by electrical waves of excitation propagating through cardiac tissue. Abnormal wave propagation may result in cardiac arrhythmias. Sudden cardiac death due to cardiac arrhythmias is among the most common causes of death in the industrialized world [1]. One of the leading causes for the onset of cardiac arrhythmias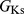 is the heterogeneity of cardiac tissue [2]–[4].

**Figure 1:**
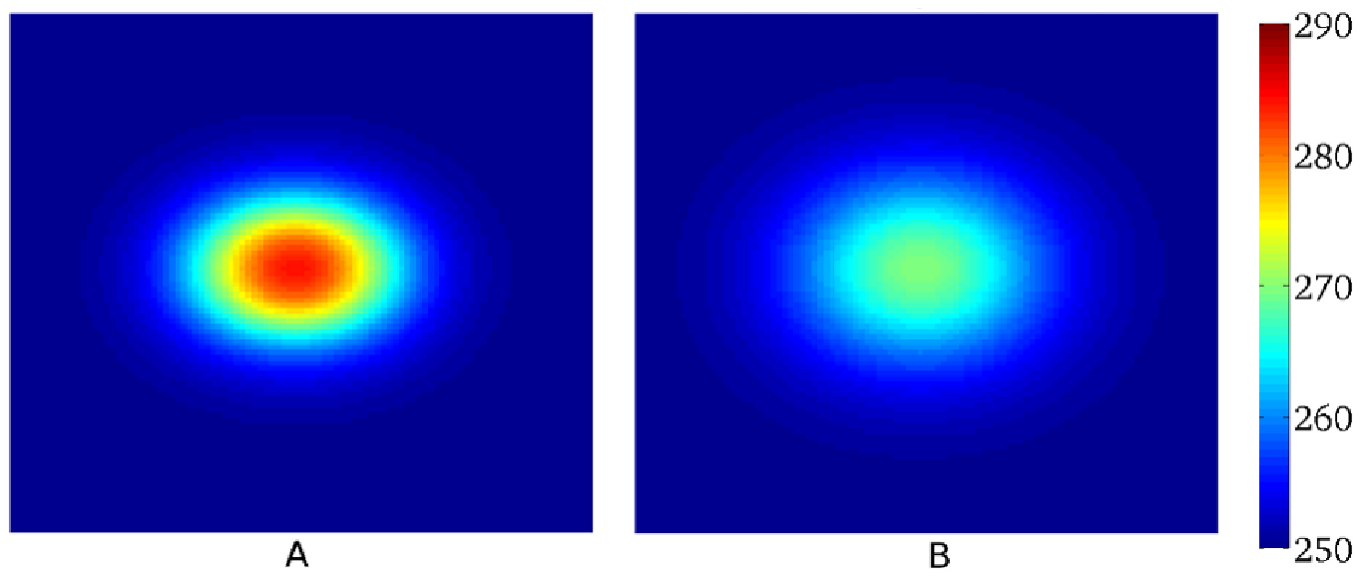
Effect of cell coupling on APD distribution simulated in a human cardiac tissue model. A: APD distribution in cardiac tissue simulated numerically in a human cardiac tissue model (9) after simultaneous excitation of all cells, to avoid effects resulting from wave propagation [6]. B: APD distribution after input of the measured data into the tissue model. Heterogeneity is created by changing *G*_Ks_. Total size of the medium is 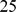
mm
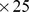
mm. Colormap shows 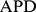 in ms.

Wave propagation in the heart is a result of succesive excitation of individual cardiac cells, which are electrically coupled to each other by gap junctions. Such electrical connectivity of the cells not only enables wave propagation, but also affects properties of the individual cells. Fig. 1A, with a maximal APD difference of 40 ms and size at 50% heterogeneity of 5 on 6 mm, shows a typical spatial action potential duration (APD) distribution similar to these measured in human heart preparations [5]. If we use these measured values as APD values at cell level, we can fit the parameters of a cell model to reproduce such APD at a given location. If we then connect these cells into the tissue model, we obtain the APD distribution as shown in Fig. 1B. We see that the shape and magnitude of the heterogeneity is substantially changed due to coupling between the cells (Fig. 1B): the amplitude of the heterogeneity in Fig. 1B is almost twice as small as the one in Fig. 1A. Alternatively, if one uses a patch clamp procedure and measures properties of various types of uncoupled cardiac cells, it is not clear which heterogeneity will be produced by these cells if they are coupled to tissue. Therefore, the question how to recover real heterogeneity from tissue level experiments and how heterogeneity at cell level manifests itself at tissue level is very important both for theoretical and experimental work.

In this article we show that such electrotonic effects on APD heterogeneity can be characterized by a linear approach using Gaussian functions fits. In particular, we show that APD at tissue level can be found by a convolution of APD distribution at cell level with a bell-shaped Gaussian function (forward problem). Convolutions involving Gaussian functions are among the most studied in mathematics, and applied to various physical, chemical and biological phenomena. Using this formalism, we are able to solve not only the forward problem (i.e. to find APD at tissue level from known APDs of individual cardiac cells), but also start developing an approach to solve the inverse problem (namely to find properties of individual cells from measurements at the tissue level). We illustrate it on several examples.

## Results

**Figure 2:**
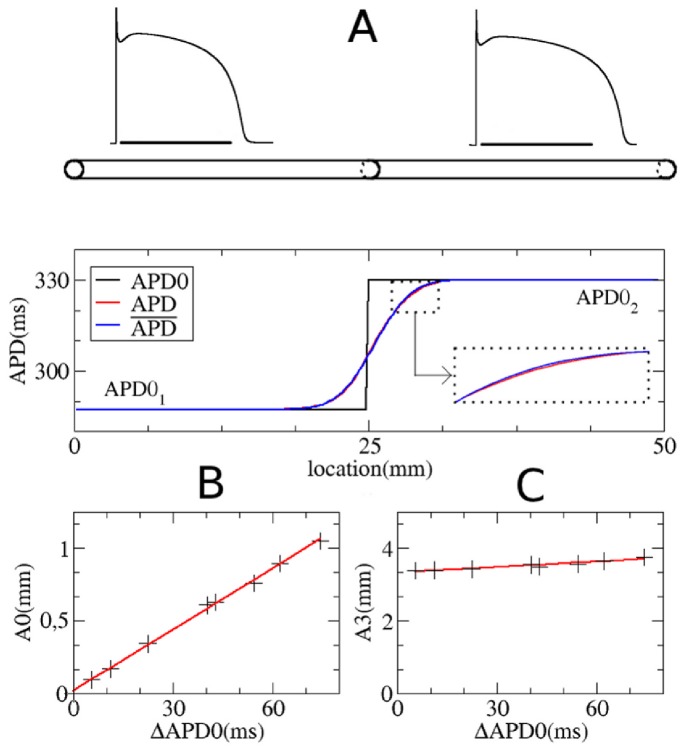
Electrotonic effect for a stepwise heterogeneity in a cable. A: Spatial distribution of 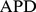 in a fiber with stepwise heterogeneity. Computations using TP06 model in a fiber of 50 mm long. The upper pannel shows action potential shapes in the uncoupled system. The black line under both action potentials represents a time interval of 300 ms. The bottom pannel shows 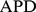 distribution: black 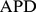 in the uncoupled system, red 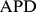 in the coupled system and blue the 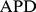 obtained via Eq.(1). B: Plot of 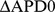 versus 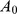. C: Plot of 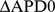 versus 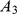. B and C: best linear fit through these points are shown in red. 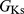 in the left part of the fiber was 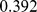
nS/pF. Other degrees of heterogeneity are obtained by changing 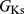. In general, for a heterogeneity of 10 ms it was necessary to change 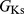 by 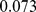
nS/pF.

To establish a proper description of the heterogeneity, we first considered the simplest type of heterogeneity: a stepwise heterogeneity in a cable (see Fig. 2A).

We excited all points of the cable simultaneously and calculated the spatial APD distribution. It is seen in Fig. 2A that, due to electrotonic effects, the stepwise heterogeneity becomes spatially distributed with a characteristic wavelength of around 3.5 mm. After trying several types of sigmoidal functions, we found that an almost perfect fit of spatial APD distribution can be obtained using the error function, which is the antiderivative of the Gaussian function. Indeed, if we use for the stepwise heterogeneity in Fig. 2A:

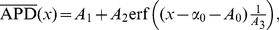
(1)with 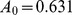
mm
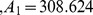
ms
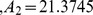
ms, 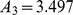
mm and the location of the heterogeneity 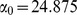
mm, then the exact solution (red line in Fig. 2A) and the fit (blue line) are almost indistinguishable from each other with a relative error less than 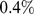.

Note that the parameters 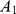 and 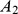 in Eq.(1) are obviously connected to the APD in the uncoupled system (APD0) from Fig. 2A as 
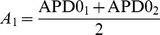
 and 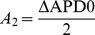 with 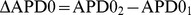. The parameter 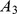 gives the spatial distribution, and in our case is 3.497 mm; the parameter 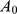 indicates some additional shift, which in our case is 0.631 mm.

By varying 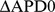, we studied how the parameters 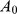 and 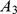 of our fit depend on the degree of heterogeneity. We found that parameter 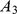 showed only minimal dependency on 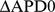. Indeed, in Fig. 2C we see that by changing 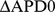 13.3 fold, 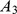 changes just by 10.8%. Furthermore, if we put 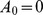, in (1), our curve is shifted to the left, and gives us a maximal error of around 2ms. However, for small values of 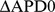, which will be the most important for us in the future 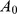 is small. For example, for 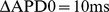, 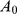 is of the order of 0.15 mm, which is approximately 20 fold less than the characteristic space constant 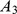. Therefore, we can conclude that with a high degree of accuracy we can assume that the parameters 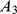 and 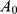 do not depend on 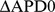.

**Figure 3:**
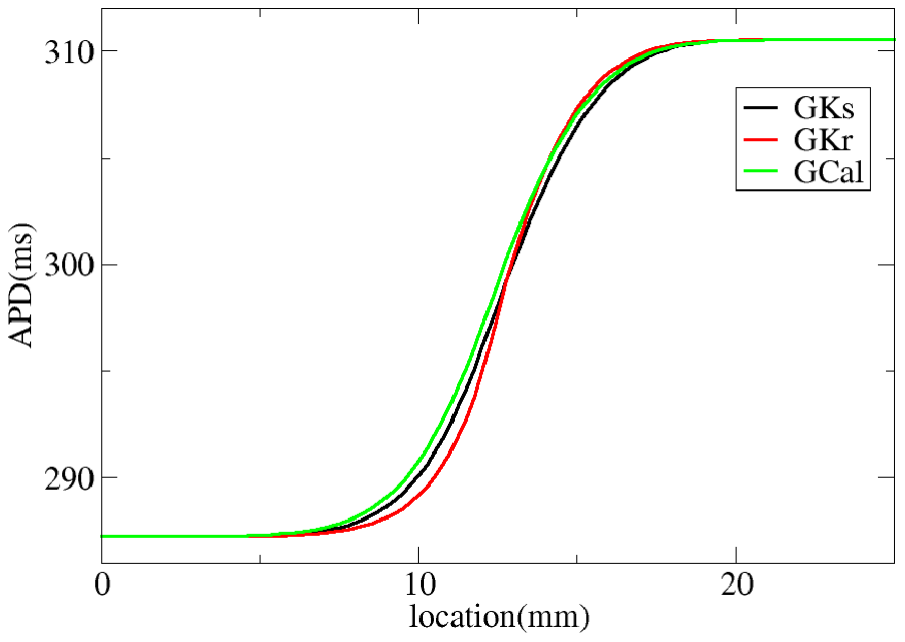
Electrotonic interaction for different parameters. In black, red and green, heterogeneity was created by changing, respectively, 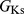, 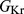 and 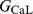. In the left part of the fiber 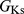 = 0.392 nS/pF; 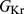 = 0.153 nS/pF and 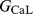 = 
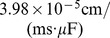
. For a heterogeneity of 10 ms it was necessary to change 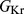 and 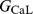 with respectively 0.048 nS/pF and 7.4
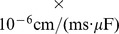
.

In Fig. 2 we studied a heterogeneity obtained by changing the 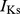 current. Besides 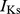, other ionic currents such as 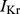 and 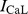 have important influence on APD. To find possible effect of other ionic currents on the electrotonic interaction we study a stepwise heterogeneity as in Fig. 2A, but with a heterogeneity now obtained by changing 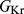, respectively 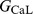 (Fig. 3). We see that the electrotonic effect in our model does not depend on ionic current used to induce tissue heterogeneity. In particular, in all cases the fit of Eq.(1) works good and we have 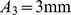, for 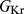 induced heterogeneity, and 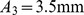 for 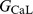 or 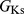 induced heterogeneity. Furthermore, we find that the parameter 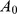 is also small. For 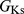, 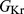 and 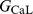, we find for 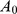 respectively 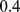, 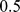 and 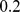 mm.

Overall we can conclude that our problem of dependency of 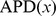 on 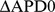 can thus with high accuracy be considered as linear. This allows us to formulate an approach for finding 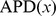 not only for a stepwise heterogeneity, but for a heterogeneity of any sharp 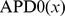.

Thus, as for a linear problem, any heaviside-like heterogeneity will generate an APD distribution given by (1), this yields that the expected 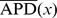 will be given by:
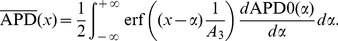
(2)


Integrating by parts yields
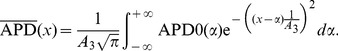
(3)


The observed distribution 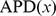 can thus be found as a convolution of APD0 with a Gaussian function.

Let us extend this formula to 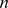 dimensions and to the general anisotropic case, as in Eq.(9). First, note that if we consider a 1D system with a constant diffusion coefficient 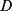, then due to spatial scaling, 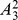 is proportional to 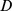 and based on our simulation we can write for an arbitary 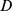 that 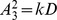, where 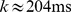. Similar considerations for a 2D case with the fibers directed along the X axis and diffusivities in the 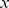 and 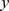 directions given by 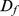 and 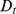 yield a Gaussian kernel 
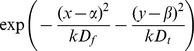
. Note, as in this case 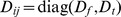, this expression can be rewritten as 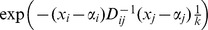, where 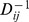 is the inverse of 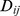. For an arbitrary fiber orientation, we can proceed to a local coordinate system aligned with the fibers, in which, as in the previous case, the diffusion tensor will be diagonal. Direct calculation shows that the general case will simply result in transformation of a diagonal matrix to a general non-diagonal matrix 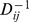, which is the inverse of the diffusivity matrix 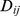 from Eq. (9), but the form of the expression will be unchanged. Thus, in 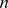 dimensions, in presence of anisotropy given by a constant matrix 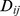, or 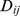 slowly varying in space, formula (3) will be given by
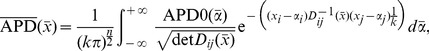
(4)with 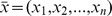, 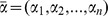. Note that, although this formula is formally valid only for a matrix 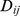 which is constant in space, it is reasonable to assume that it will be also valid for fibers for which the orientation changes slowly in space, as the Gaussian function in Eq.(4) is exponentially localized in space. Possible extensions of this formula to a general curved space will be studied in a subsequent publication.

**Figure 4:**
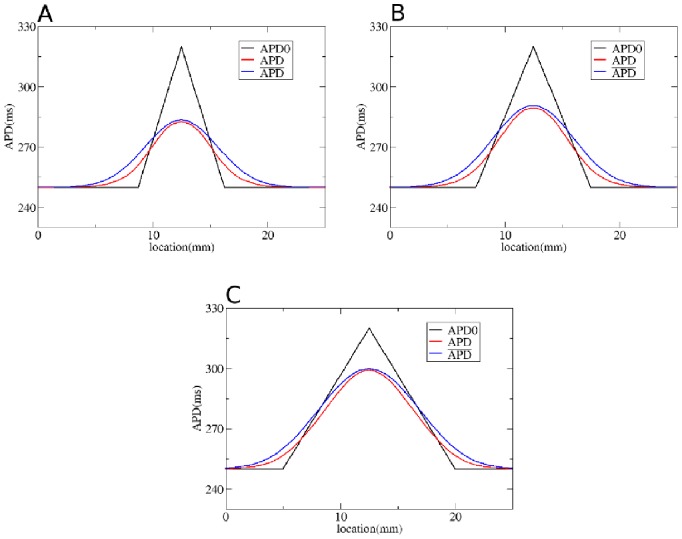
Solution of the forward problem for heterogeneity created by changing 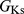. In A, B and C: in black APD0, in red APD, and in blue the predicted APD via Eq. (3) with 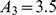 mm and 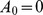 mm. Parameters of the model in A are: 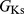 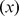 = 0.72 nS/pF if 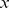 is smaller than 9 mm; 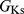 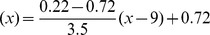 nS/pF if 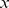 is between 9 mm and 12.5 mm; 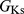 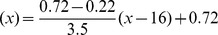 nS/pF if 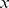 is between 12.5 mm and 16 mm; 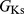 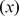 = 0.72 nS/pF if 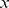 is larger than 16 mm. In B: 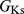 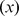 = 0.72 nS/pF if 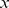 is smaller than 7.5 mm; 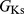 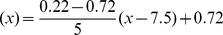 nS/pF if 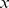 is between 7.5 mm and 12.5 mm; 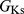 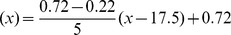 nS/pF if 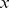 is between 12.5 mm and 17.5 mm; 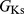 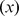 = 0.72 nS/pF if 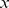 is larger than 17.5 mm. In C: 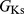 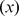 = 0.72 nS/pF if 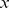 is smaller than 5 mm; 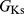 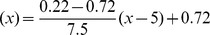 nS/pF if 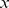 is between 5 mm and 12.5 mm; 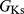 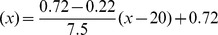 nS/pF if 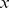 is between 12.5 mm and 20 mm; 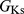 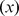 = 0.72 nS/pF if 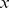 is larger than 20 mm.

**Figure 5:**
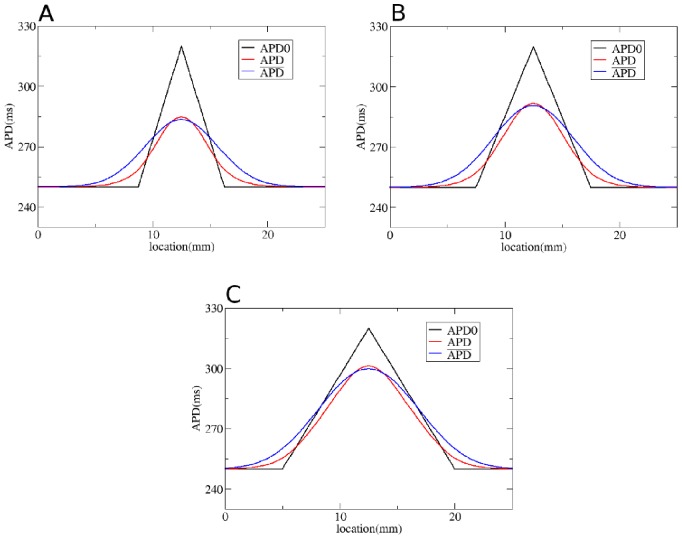
Solution of the forward problem for heterogeneity created by changing 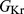. In A, B and C: in black APD0, in red APD, and in blue the predicted APD via Eq. (3) with 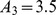 mm and 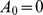 mm. Parameters of the model in A are: 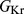 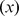 = 0.35 nS/pF if 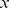 is smaller than 9 mm; 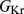 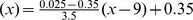 nS/pF if 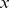 is between 9 mm and 12.5 mm; 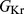 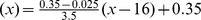 nS/pF if 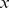 is between 12.5 mm and 16 mm; 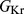 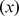 = 0.35 nS/pF if 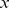 is larger than 16 mm. In B: 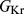 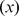 = 0.35 nS/pF if 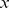 is smaller than 7.5 mm; 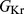 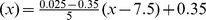 nS/pF if 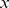 is between 7.5 mm and 12.5 mm; 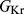 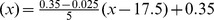 nS/pF if 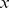 is between 12.5 mm and 17.5 mm; 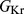 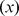 = 0.35 nS/pF if 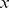 is larger than 17.5 mm. In C: 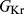 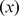 = 0.35 nS/pF if 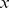 is smaller than 5 mm; 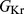 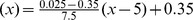 nS/pF if 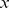 is between 5 mm and 12.5 mm; 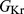 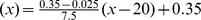 nS/pF if 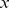 is between 12.5 mm and 20 mm; 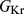 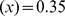 nS/pF if 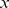 is larger than 20 mm.

**Figure 6:**
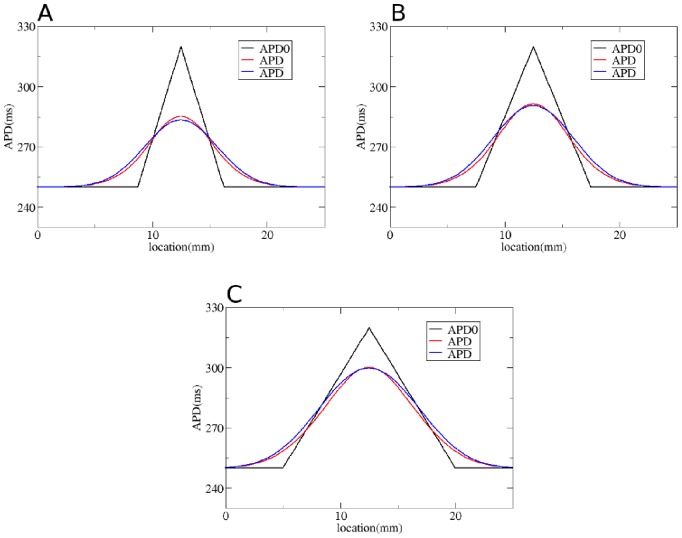
Solution of the forward problem for heterogeneity created by changing 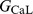. In A, B and C: in black APD0, in red APD, and in blue the predicted APD via Eq. (3) with 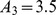 mm and 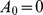 mm. Parameters of the model in A are: 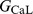 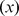 = 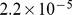 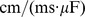 if 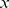 is smaller than 9 mm; 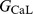 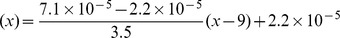 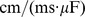 if 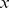 is between 9 mm and 12.5 mm; 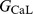 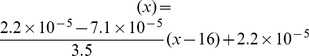 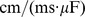 if 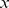 is between 12.5 mm and 16 mm; 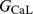 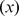 = 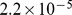 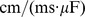 if 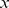 is larger than 16 mm. In B: 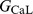 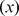 = 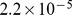 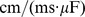 if 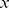 is smaller than 7.5 mm; 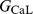 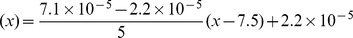 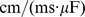 if 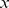 is between 7.5 mm and 12.5 mm; 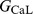 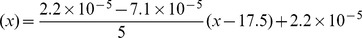 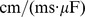 if 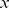 is between 12.5 mm and 17.5 mm; 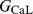 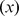 = 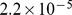 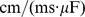 if 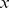 is larger than 17.5 mm. In C: 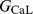 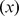 = 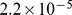 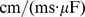 if 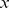 is smaller than 5 mm; 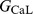 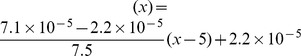 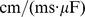 if 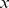 is between 5 mm and 12.5 mm; 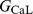 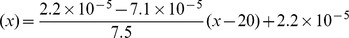 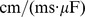 if 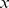 is between 12.5 mm and 20 mm; 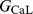 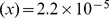 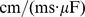 if 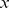 is larger than 20 mm.

**Figure 7:**
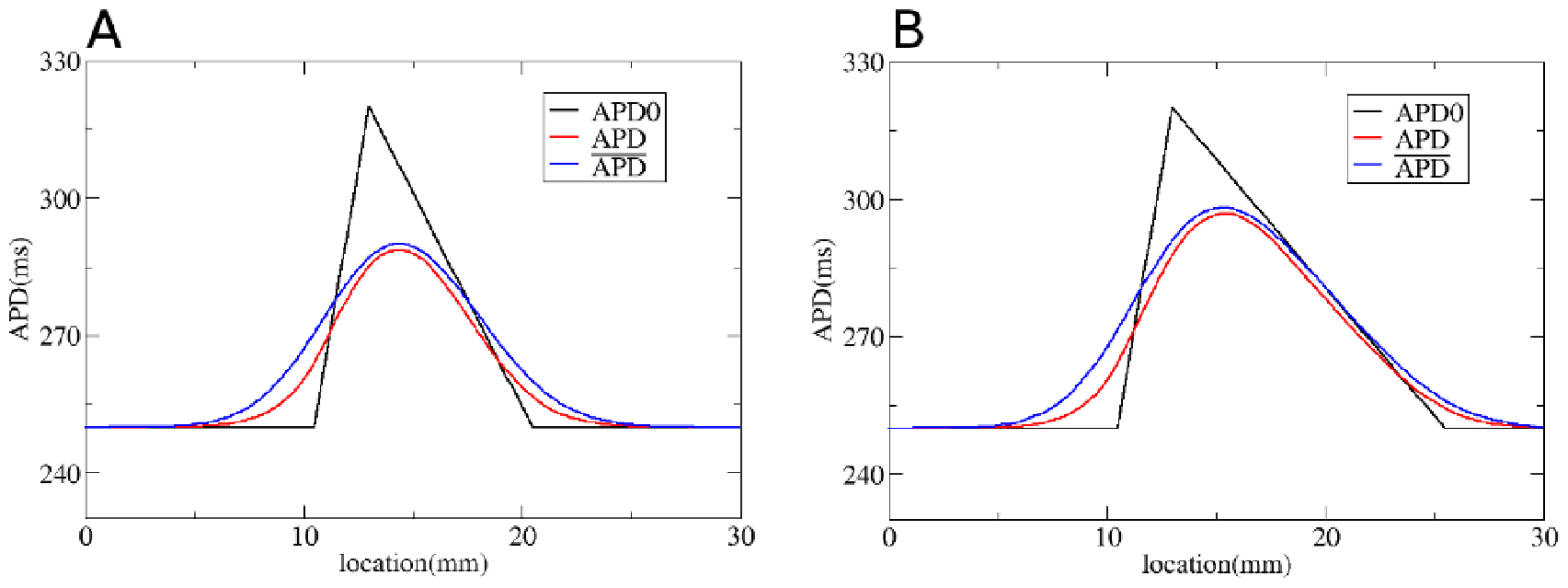
Solution of the forward problem for an asymmetric heterogeneity created by changing 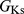. In A and B: in black APD0, in red APD, and in blue the predicted APD via Eq. (3) with 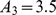 mm and 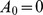 mm. Parameters of the model in A are: 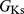 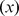 = 0.72 nS/pF if 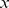 is smaller than 10.5 mm; 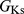 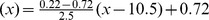 nS/pF if 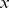 is between 10.5 mm and 13 mm; 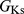 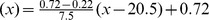 nS/pF if 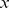 is between 13 mm and 20.5 mm; 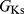 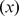 = 0.72 nS/pF if 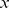 is larger than 20.5 mm. In B: 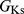 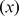 = 0.72 nS/pF if 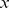 is smaller than 10.5 mm; 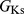 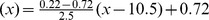 nS/pF if 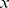 is between 10.5 mm and 13 mm; 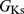 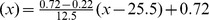 nS/pF if 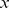 is between 13 mm and 25.5 mm; 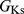 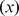 = 0.72 nS/pF if 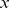 is larger than 25.5 mm.

We first test our method by considering gradients in APD with different steepness obtained by changing 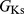 (Fig. 4), 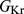 (Fig. 5) and 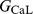 (Fig. 6). We use Eq.(3) to predict APD duration in the coupled system and observe that our method works good for the heterogeneities induced by 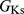 and 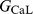. For the heterogeneity induced by 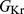 (Fig. 5), we see some deviations from the predicted values, especially for steep heterogeneities. However, we see that in all situations our method predicts the maximal value of APD in the coupled system, which is important for characterization of the extent of heterogeneity. We also did two simulations for an asymmetric heterogeneity (see Fig. 7). We see that our method also works good in that case.

**Figure 8:**
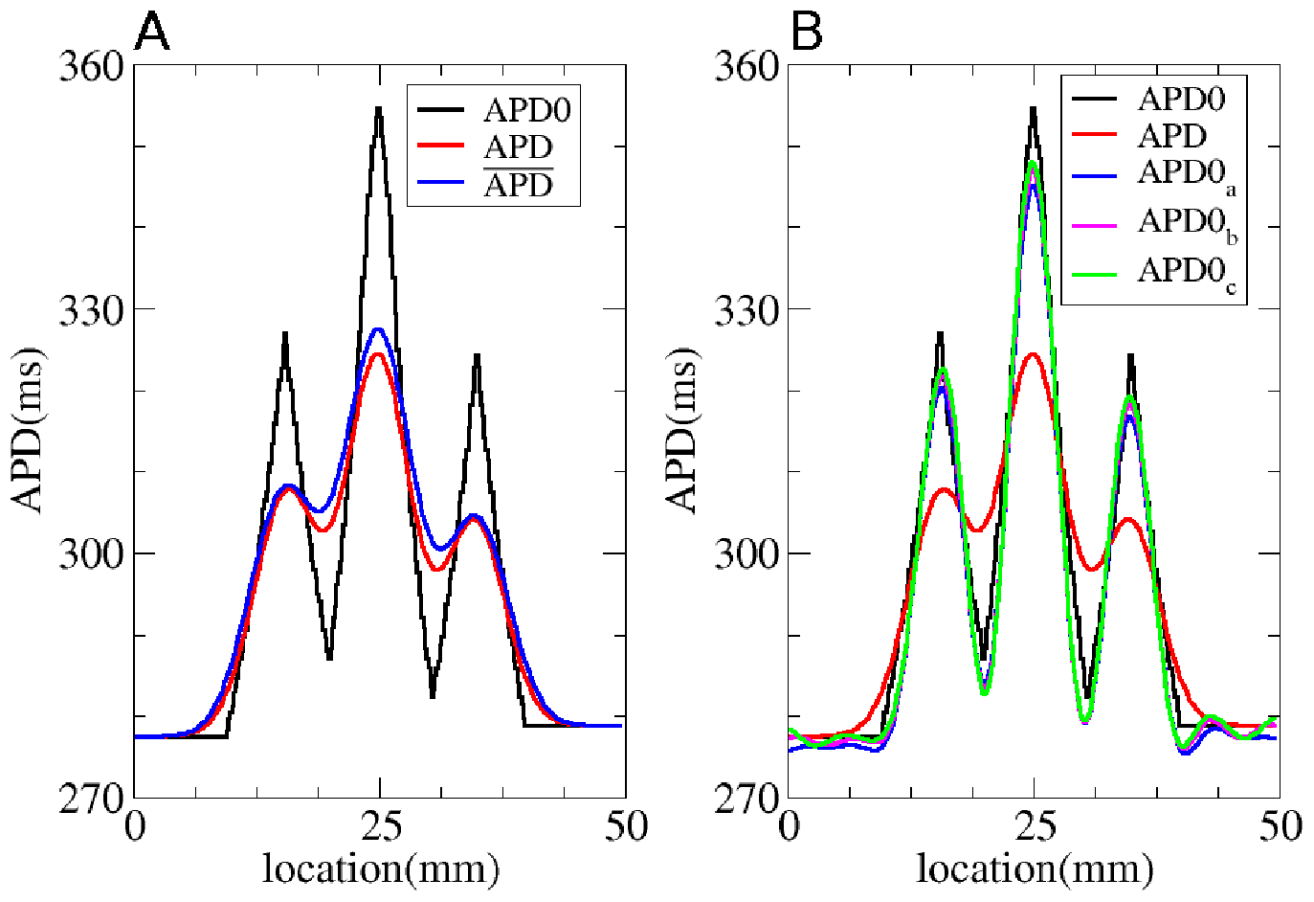
Solution of the forward and inverse problem in 1D. A: The solution of the forward problem in 1D. In black the 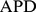 in the uncoupled system, in red 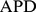 in the coupled system and in blue the 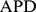 obtained via Eq. (3) with 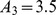 mm and 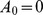 mm. B: The solution of the inverse problem in 1D. Black represents 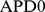 and red 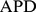 for the coupled system. 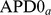 (blue), 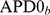 (pink) and 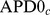 (green) display the predicted 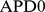 values for regularization parameter 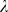 equal to 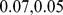 or 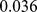.

Next, we test our method for a more complex 1D heterogeneous APD0 distribution (Fig. 8A). Heterogeneity is, in this case, created by changing 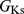. We see that Eq.(3), with 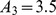 mm gives a good prediction for the observed APD distribution, with a maximal error of 3 ms. To quantify how well our approach predicts the electrotonic effect, we compared the measured electrotonic effect (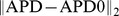, using the L2 norm) with the predicted electrotonic effect (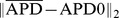) as 
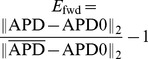
. For Fig. 8A, we obtained 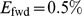.

**Figure 9:**
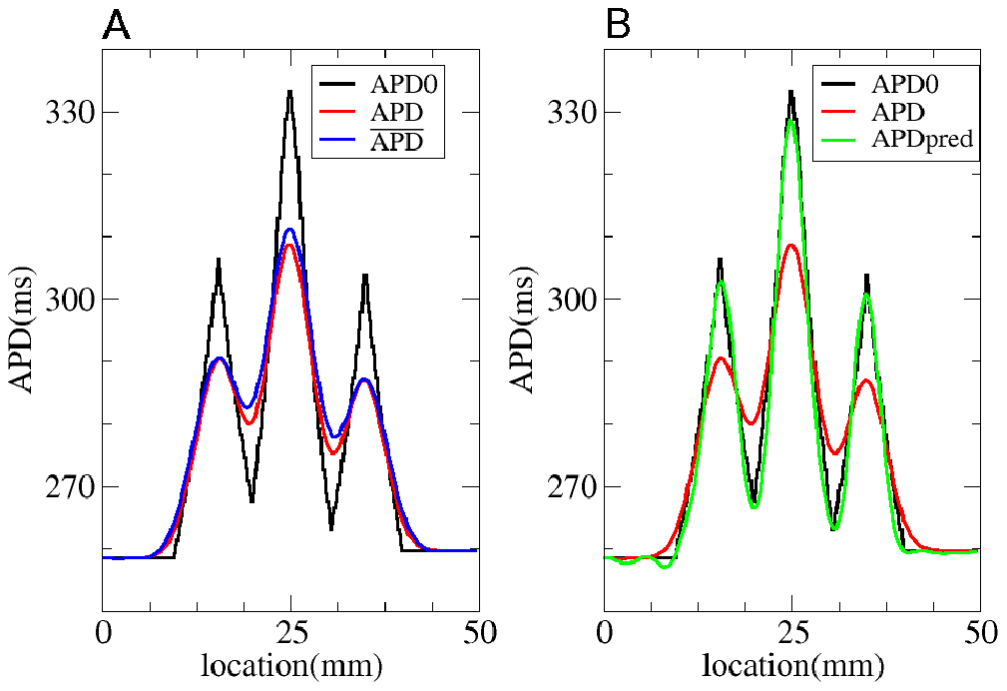
Solution of the forward and inverse problem in 1D with APD measured at 50%. A: The solution of the forward problem in 1D. In black the 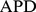 in the uncoupled system, in red 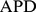 in the coupled system and in blue the 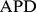 obtained via Eq. (3) with 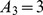 mm and 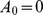 mm. B: The solution of the inverse problem in 1D. Black represents 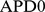 and red 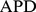 for the coupled system. Green shows the predicted 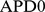 for regularization parameter 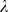 equal to 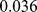.

We have also checked if our method works for the electronic effects for APD measured at 50% repolarization level (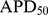). As electrotonic effects depend on the level at which we perform a measurement [6], we recalculated 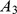 for this case, which was found to be 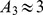 mm. We performed the same simulations as those in Fig. 8A for 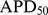. We see (Fig. 9A) that Eq.(3), gives a good prediction for the observed 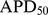 distribution, with a maximal error of 3 ms and 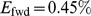.

We performed the same analysis in 2D for a diffusivity matrix 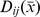 which changes slowly in space using (4). For this, we supposed that the fibers lay along parabolas 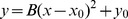, with 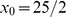 mm, varying 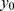 and a fixed shape parameter 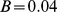 1/mm. This gives us the local fiber direction
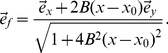
(5)


**Figure 10:**
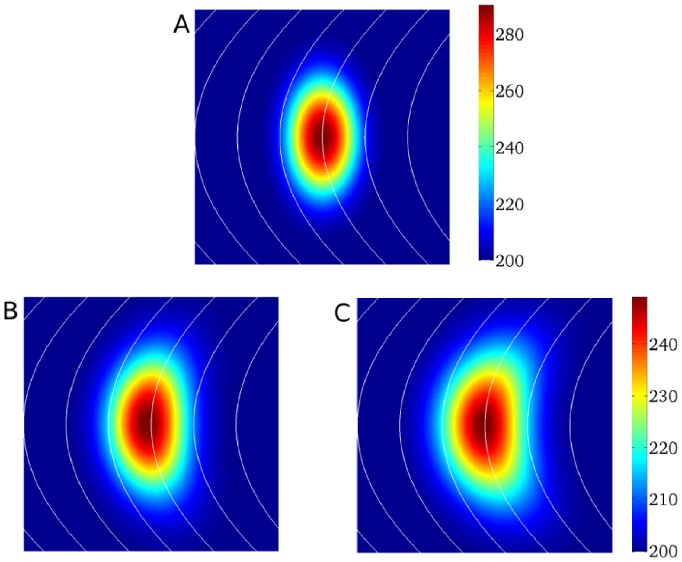
Solution of the forward problem in 2D for a curved space. A: APD0 distribution. B: APD distribution after input of APD0 in a human cardiac tissue model. C: Predicted APD distribution obtained using formula (4) from main text. Fiber direction is drawn in white lines.

Thus for the diffusivity matrix 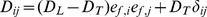, we obtain
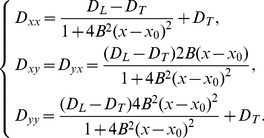
(6)with in our case 
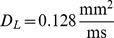
, and 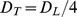. In Fig. 10A we show APD0, in Fig. 10B measured APD and in Fig. 10C the predicted values 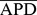. Here, we found a maximal error of 6 ms, and 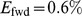. We can thus conclude that our forward method for calculating APD by convoluting APD0 with a Gaussian function produces accurate results.

**Figure 11:**
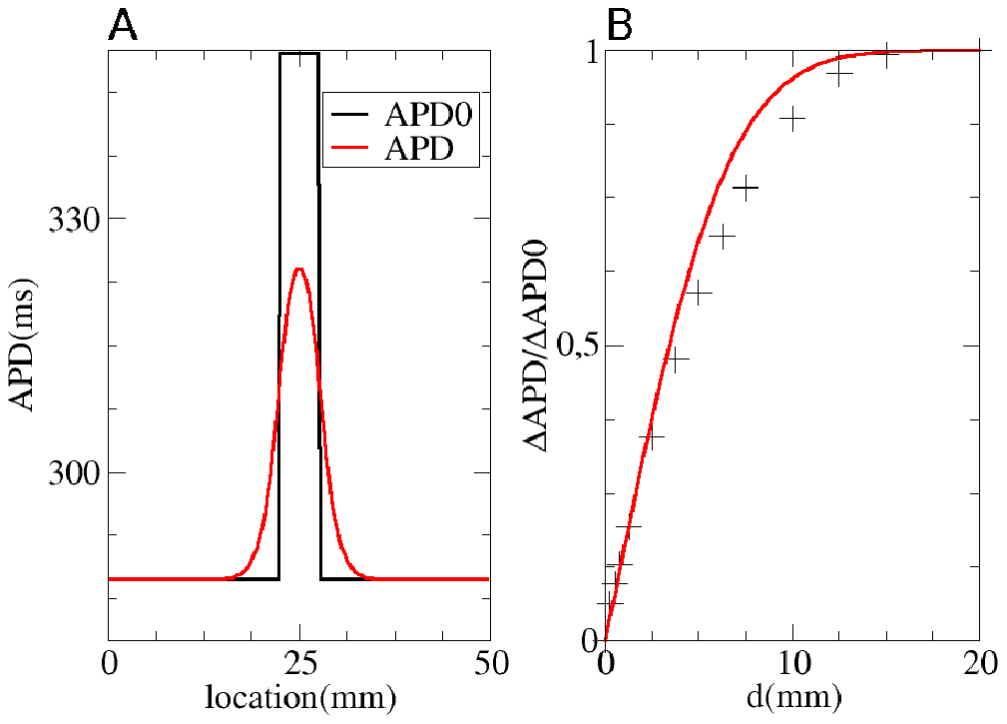
Analytical estimate of electrotonic effect. A: In black APD0, in red APD. B: In red Eq. (8). The '
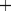
's are the values obtained via simulations.



The proposed approach can also be used for finding analytical estimates of electrotonic effects. Let us apply it to estimate dependence of electronic effects on the size of heterogeneity. For this, consider a block of size 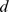 of tissue with a longer APD (Fig. 11A) and compare maximum of APD in coupled system relative to the maximum in the uncoupled system. For such step-wise distribution, the APD0 integral (2) can be evaluated explicity, yielding for the maximal value of APD:
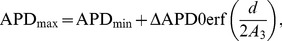
(7)or thus

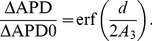
(8)This curve is plotted in red in Fig. 11B. We also plot the values we obtained via simulations. We see a good correspondence of numerical and analytical estimates. The correspondence is perfect for a small and large thickness of the heterogeneity, although in the intermediate range, we have some deviations. We also see that the electrotonic effects are substantially affected by heterogeneity size. Indeed, we see that if the heterogeneity is 1–2 mm, the value of the heterogeneity measured in tissue experiments will differ from the real heterogeneity 2–5 fold.

Because the forward problem can be written in the standard form (3) and (4), it can also be used to solve the inverse problem, i.e. predict APD0 from a given APD. Standard approaches to solve inverse problems of this type use regularization methods [7]. Here, we will use the most common and well-known form of regularization, namely Tikhonov regularization [8]. The regularized solution via Tikhonov regularization is given by.
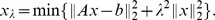



With 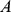 the Gaussian kernel, 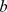 the APD distribution in the coupled system and 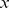 the unknown APD distribution in the uncoupled system. Thus, 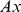 indicates the solution of the forward problem. The amount of regularization is controlled by the regularization parameter 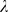, which depends on the problem itself. Larger values of 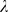 produce increasingly smoother solutions, while for small 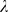 values the inverse solution is less stable. We note that properly choosing 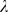 is a common problem of all inverse problems. There is basically only one established approach, called the Morozov discrepancy principle [7]; however, it does not always produce a suitable value for 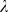. In the present work, we manually adapted 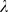 until the solution to the inverse problem appeared as regular. Our criterion for this was: a small oscillatory component.

**Figure 12:**
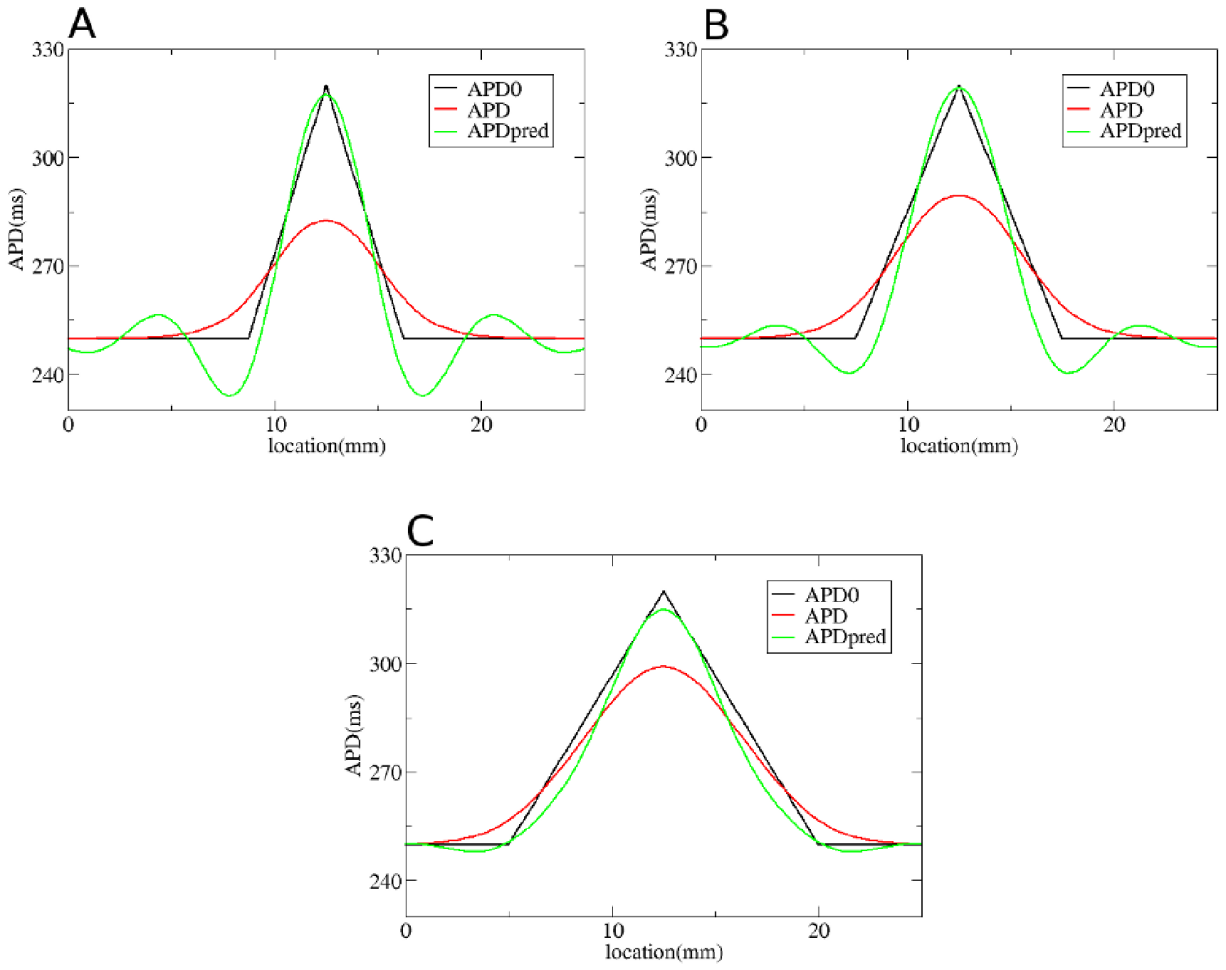
Solution of the inverse problem for heterogeneity created by changing 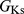. In A, B and C: in black APD0, in red APD, and in green the predicted APD0 via Tikhonov regularization. Parameters of the model for A, B and C are the same as in Fig. 4. Regularization parameter is 0.05.

**Figure 13:**
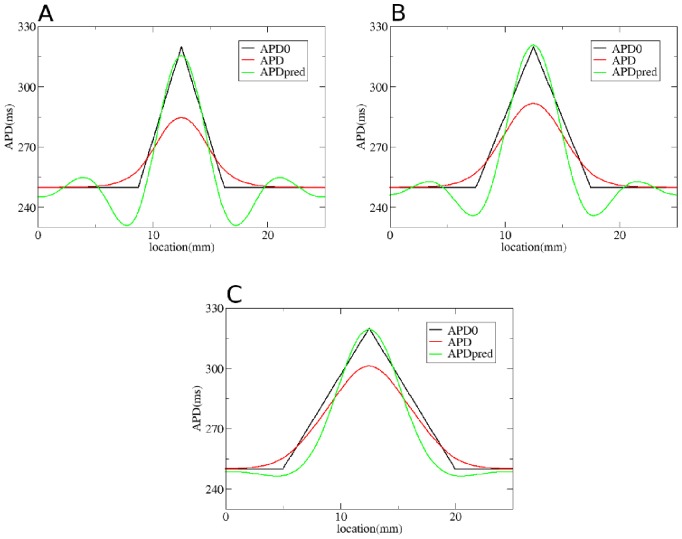
Solution of the inverse problem for heterogeneity created by changing 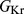. In A, B and C: in black APD0, in red APD and in green the predicted APD0 via Tikhonov regularization. Parameters of the model for A, B and C are the same as in Fig. 5. Regularization parameter is 0.09.

**Figure 14:**
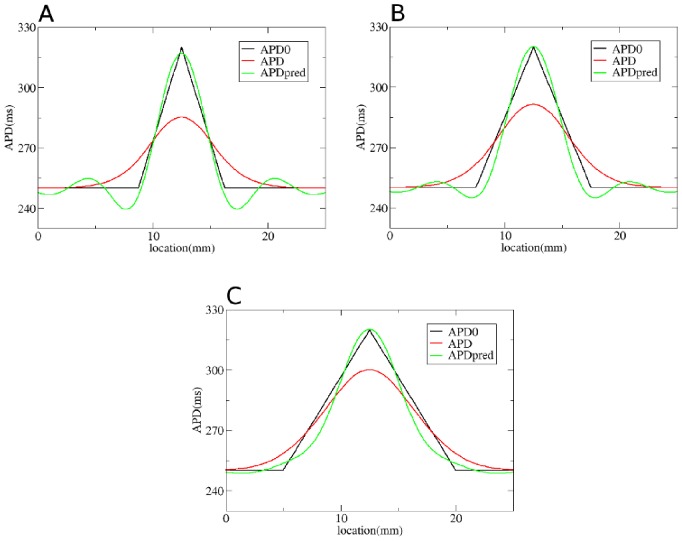
Solution of the inverse problem for heterogeneity created by changing 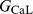. In A, B and C: in black APD0, in red APD and in green the predicted APD0 via Tikhonov regularization. Parameters of the model for A, B and C are the same as in Fig. 6. Regularization parameter is 0.05.

**Figure 15:**
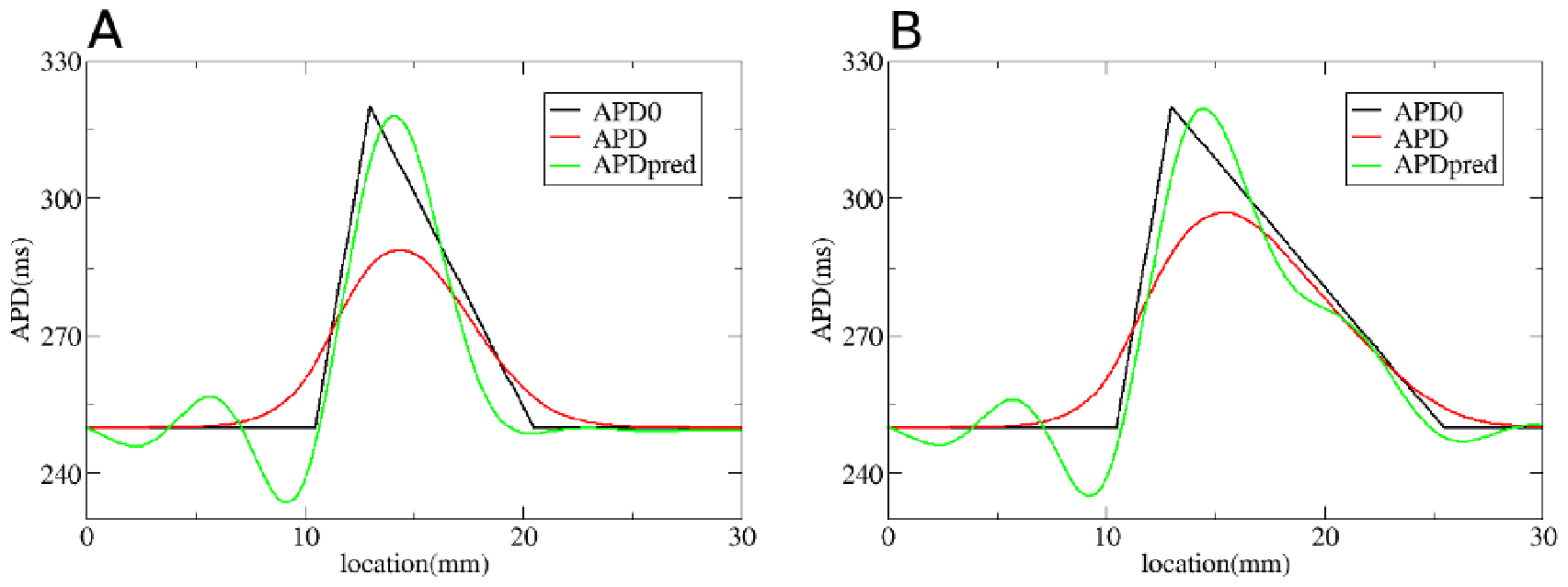
Solution of the inverse problem for an asymmetric heterogeneity created by changing 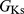. In A and B: in black APD0, in red APD and in green the predicted APD0 via Tikhonov regularization. Parameters of the model for A, B and C are the same as in Fig. 7. Regularization parameter is 0.05.

To solve the inverse problem in 1D via Tikhonov regularization, we used the algorithms available at [8]. As for the forward problem, we first consider simple gradients in Fig. 12, 13 and 14. Heterogeneities are the same as in Fig. 4, 5 and 6, and thus obtained by changing, respectively, 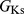, 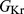 and 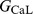. We see that for intermediate and shallow heterogeneities, we can reconstruct APD0 with reasonably high accuracy (Fig. 12B, C; 13B, C and 14B, C) especially the maximal value of APD0. We see that in all solutions, there is an oscillatory component present which increases with the increase of steepness of the gradient. This causes substantial errors for the steepest heterogeneity (Fig. 12A, Fig. 13A and Fig. 14A). We also applied our method for the two asymmetric heterogeneities as in Fig. 7. In Fig. 15, we see that we can reconstruct APD0 in both cases. Again, we observe an oscillatory component, especially for the steep part of the heterogeneity.

Now we illustrate our method on a more complex APD0 distribution. We consider the same APD0 distribution as in Fig. 8A. In Fig. 8B, we reconstruct the initial APD0 distribution (black), based on the measured APD distribution (Fig. 8B, red). Three reconstructions are shown, for various values of the Tikhonov parameter 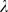. The maximal absolute errors for 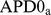, 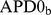, 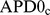 are respectively 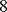 ms, 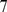
ms and 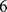 ms. In the same way as for the forward problem, we can quantify how well our approach reproduces the electrotonic effects by calculating 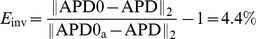. For 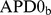, 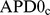, we find respectively 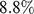 and 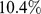. Thus, we observe that this method works well and that we can recover the APD0 distribution in the uncoupled system.

Fig. 9B illustrates that the method also works good for 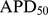. We see that the predicted APD0 (green) is close to the initial APD0 distribution. The maximal absolute error is 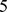 ms and 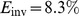, which is close to that for the APD distribution measured at 80% repolarization level.

**Figure 16:**
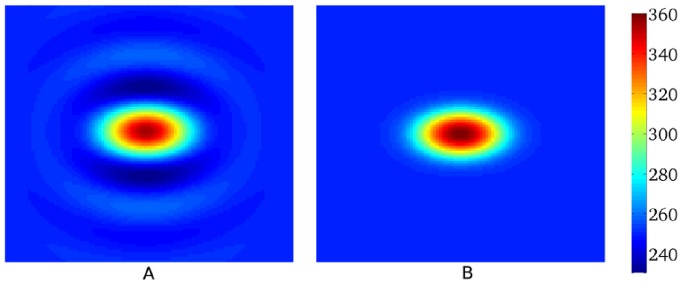
Solution of the inverse problem in 2D. A: The predicted 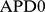 values for a measured 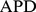 distribution given by Fig. 1A. Regularization parameter 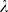 is 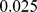. B: The exact solution.

To solve the inverse problem in 2D via Tikhonov regularization, we used the algorithms from [9]. To test our method in 2D, we used the dataset from Fig. 1A, and quantified the real heterogeneity via the solution of the inverse problem. We obtain the solution shown in Fig. 16A. In Fig. 16B we plot the exact APD0 distribution used in our model to obtain Fig. 1A. In this way, we see that our inverse solution properly recovers the underlying heterogeneity. As in 1D, we calculate 
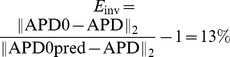
. In particular, the most important parameter: the maximal value of APD0 in the inverse solution is 355 ms, while the exact value is 360 ms. The characteristic width at 50% of heterogeneity in the inverse solution is 2 mm in the vertical and 3 mm in the horizontal direction, while the exact values are 2 mm and 3.25 mm. However, we also see that, as in 1D, the inverse solution has a damped oscillatory component in certain directions, with amplitude up to 20 ms. This component is absent in the original APD0 distribution. Note, however, that we applied the simplest method for solution of the inverse problem here, so it can certainly be improved. For, the inverse problem given by Eq. (4) is one of the most studied inverse problems in applied mathematics. Thus it should be possible to suppress the oscillatory component by choosing a proper formulation, which we intend to do in the future.

## Discussion

In this paper we have shown that the electrotonic effects in heterogenous cardiac tissue can with good accuracy be treated using a linear Green's function approach. Interestingly, a good approximation for the Green's function is given by a Gaussian kernel. This relates our problem to one of the most studied classical problems in science and engineering arising from the diffusion equation, such as mass and heat transfer, image processing, light scattering etc.

We have shown that even the most simple and straightforward approaches for the forward and inverse problems produce promising results, which opens possibilities for the application of this approach to computational studies as well as to experimental research. Regarding the forward problem, we showed that our method works good, and that, even for steep gradients, we can predict the maximal value of APD in the coupled system, which is important for characterization of the extent of heterogeneity. Our solution of the inverse problem is also promising for non steep gradients. However, for steep gradients we have an additional oscillatory component outside the heterogeneity which does not allow us to determinate the maximal amplitude with sufficient degree of certainty. This is a well known feature of the inverse solutions. The solution may certainly be improved by using, for example, different norms of Tikhonov regularization etc. This subject requires additional specific investigation and the development of non-standard software, which is outside the scope of this manuscript.

Application of the approach to computational studies for both forward and inverse problems is straightforward. To accomplish this, one just needs to determine the parameter 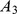 for the Green's function, which can easily be done by direct numerical computations similar to those shown in Fig. 2.

Applying the approach to experimental studies is more difficult and several important issues still need to be investigated. The most important of them is to determine the space constant of the Green's function. The best approach here would be direct measurement of spatial distribution of repolarization in tissues with known heterogeneity. This heterogeneity might be static or dynamic (e.g. by local injection of currents into cardiac cells). Also, computational [6] and recent experimental data [10] shows that the extent of electrotonic effects depends on the shape of the action potential. It would thus be interesting to investigate the possibility to determine the space constant of the Green's function from measured action potential shapes.

**Figure 17:**
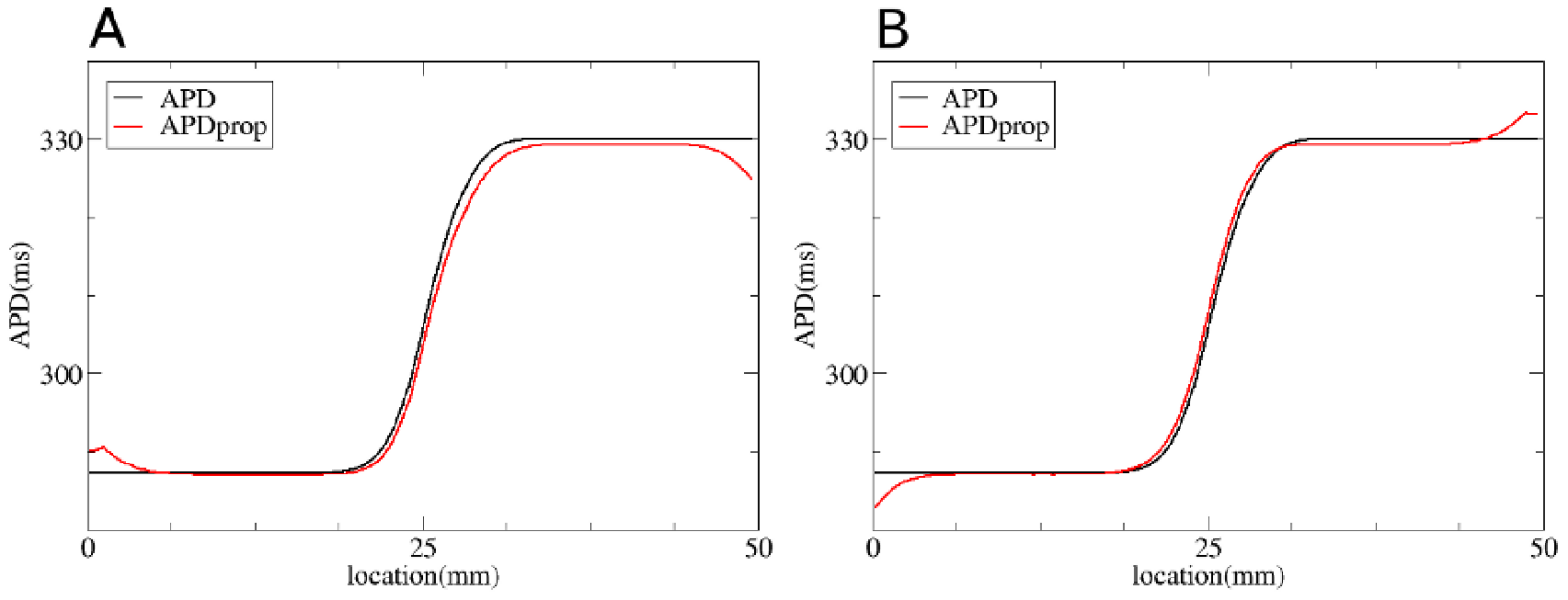
Effect of wave propagation in 1D. A: In black APD distribution obtained via simultaneous stimulation of all cells. In red the APD distribution obtained by wave propagating from the left boundary. B: Same as A, but now in red the APD distribution obtained by wave propagating from the right boundary.

**Figure 18:**
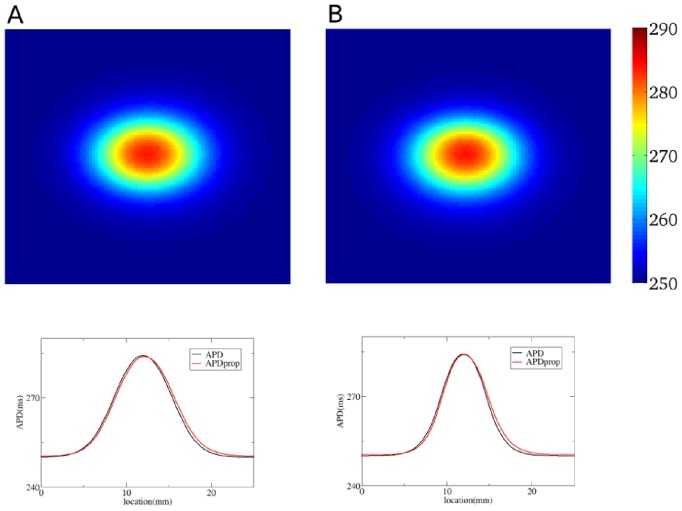
Effect of wave propagation in 2D. A: Upper panel: APD distribution obtained by wave propagating from the left boundary. Lower panel: APD distribution along the horizontal line through the center. In black for simultaneous stimulation of all cells as in Fig. 1A. In red for stimulation from the left boundary. B: Upper panel: APD distribution obtained by wave propagating from the upper boundary. Lower panel: APD distribution along the vertical line through the center. In black for simultaneous stimulation of all cells. In red for stimulation from the upper boundary.

As in [6], we studied electrotonic effects by simultaneous stimulation of all cells. In such an approach, effects arising from wave propagation are absent. However, this stimulation protocol is difficult to realize in experiments. To account for this shortcoming we compared several typical APD distributions obtained by this protocol with those resulting from wave propagation. In Fig. 17A and B we compare the simultaneous stimulation (black line) and stimulation by wave propagating from the left, respectively right, boundary (red line). Fig. 18A and B shows the APD profile for the same heterogeneity as shown in Fig. 1A, but obtained by wave propagating from the left, respectively right, boundary. In both cases the changes due to the different stimulation protocol are less than 1% and thus comparable with errors of the method. This causes the results obtained here to be also valid in the case where the APD heterogeneity measured from the propagating waves is used.

In this paper, we used a monodomain description of cardiac tissue, see (9). Another widely used model for cardiac tissue is a bidomain model [11]. Note, however, that in 1D, the bidomain and monodomain approaches co-inside. Therefore, all our 1D results for the forward and inverse problem will be valid for the bidomain case as well. In 2D, effects on non-equal anisotropy ratio, although essential for defibrillation problems, normally have a small effect on normal wave propagation [12]. Therefore, we expect little effect of using bidomain equations on our approach. However, it would be interesting to study it in the future.

The fact that the error function almost perfectly fits the APD profiles found, indicates that electrotonic effects are closely related to processes described by the diffusion equation. It would be interesting to investigate this similarity using analytical approaches applied to equation (9).

## Materials and Methods

### Model

In this paper we consider a monodomain description of cardiac tissue [13] which has the following form:
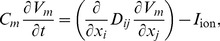
(9)where 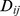 is a diffusion matrix accounting for anisotropy of cardiac tissue, 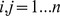, where 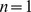 in 1D, 2 in 2D…, 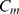 is membrane capacitance, 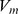 is transmembrane voltage, 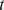 is time and 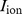 is the sum of ionic transmembrane currents describing the excitable behavior of individual ventricular cells. To represent human ventricular electrophysiological properties, we used the ionic TP06 model [14], [15]. This model provides a detailed description of voltage, ionic currents, and intracellular ion concentrations for human ventricular cells. A complete list of all equations can be found in [14], [15]. We used the 'default' parameter settings from [15] for epicardial cells. All parameter changes made to obtain tissue heterogeneity are detailed in the text.

### Numerical Methods

For 1D and 2D computations, the forward Euler method was applied to integrate (9). A space step of 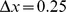 mm and a time step of 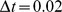 ms were used. To integrate the Hodgkin-Huxley-type equations for the gating variables of the various time-dependent currents (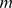, 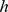 and 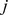 for 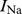; 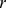 and 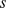 for 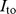; 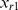 and 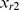 for 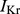; 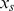 for 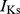; 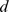, 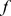, 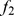 and 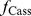 for 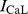), the Rush and Larsen scheme [16] was used.

### Heterogeneity

To study heterogeneity, we change the parameters 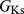, 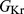 and 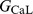 from their default values 0.392 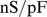, 0.153 nS/pF and 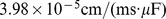 for epicardial cells in [15]. Unless otherwise stated, APD is measured at 80% repolarization level.

### Inverse Problem

The inverse problem in 1D was solved using Tikhonov's regularization method. In 2D, the inverse problem was solved using the Tikhonov image deblurring fast fourier transform algorithm. To implement this in 1D and 2D we used two Matlab packages developed by Per Christian Hansen [8], [9].

## Acknowledgments

We are thankful to Prof. Y. Kurylev for an important discussion.
